# Using vision transformers for electrographic seizure classification to aid physician review of intracranial electroencephalography recordings

**DOI:** 10.3389/fnhum.2025.1680395

**Published:** 2025-10-08

**Authors:** Muhammad Furqan Afzal, Sharanya A. Desai, Wade Barry, Thomas K. Tcheng, Jonathan Kuo, Shawna W. Benard, Christopher B. Traner, David Greene, Cairn G. Seale, Martha J. Morrell

**Affiliations:** ^1^NeuroPace, Inc., Mountain View, CA, United States; ^2^Department of Neurology, University of Southern California, Los Angeles, CA, United States; ^3^Cleveland Clinic Neurological Institute, Cleveland, OH, United States

**Keywords:** vision transformer (ViT), deep learning, electrographic seizure classification, intracranial EEG, explainability, epilepsy

## Abstract

We introduce a vision transformer (ViT)-based approach for automated electrographic seizure classification using time-frequency spectrogram representations of intracranial EEG (iEEG) recordings collected from patients implanted with the NeuroPace^®^ RNS^®^ System. The ViT model was trained and evaluated using 5-fold cross-validation on a large-scale dataset of 136,878 iEEG recordings from 113 patients with drug-resistant focal epilepsy, achieving an average test accuracy of 96.8%. Clinical validation was performed on an independent expert-labeled dataset of 3,010 iEEG recordings from 241 patients, where the model achieved 95.8% accuracy and 94.8% F1 score on recordings with unanimous expert agreement, outperforming both ResNet-50 and standard 2D CNN baselines. To evaluate generalizability, the model was tested on a separate out-of-distribution dataset of 136 recordings from 44 patients with idiopathic generalized epilepsy (IGE), achieving over 75% accuracy and F1 scores across all expert comparisons. Explainability analysis revealed focused attention on characteristic electrographic seizure patterns within iEEG time-frequency spectrograms during high-confidence seizure predictions, while more diffuse attention was observed in non-seizure classifications, providing insight into the underlying decision process. By enabling reliable electrographic seizure classification, this approach may assist physicians in the manual review of large volumes of iEEG recordings.

## 1 Introduction

Accurate seizure tracking is important for effective epilepsy management, as it supports monitoring of treatment response, detection of changes in seizure frequency, and informed clinical decision-making. However, the most commonly used method of seizure monitoring- patient or caregiver-reported seizure diaries- is often unreliable. Studies have shown that many seizures are frequently missed or inaccurately recorded (Blum et al., 1996; Kerling et al., 2006; Inoue and Mihara, 1998). As a result, clinical decisions based solely on seizure diaries may be inaccurate and could lead to suboptimal treatment strategies. These limitations highlight the need for objective tools to track changes in seizure frequency.

The NeuroPace^®^ RNS^®^ System is an FDA-approved implantable device that delivers neurostimulation and records iEEG data from patients with drug-resistant focal epilepsy (Skarpaas et al., 2019). By capturing long-term activity directly from the brain, the RNS^®^ System provides a rich source of objective information that can be invaluable in identifying seizure patterns and evaluating treatment responses (Desai et al., 2019). Despite the clinical potential of this iEEG data, its manual analysis remains a significant bottleneck. Reviewing and annotating electrographic seizure events across thousands of hours of recordings is highly labor-intensive, requiring expert epileptologists. This process is not only time-consuming and costly, but also prone to inter-rater variability and fatigue-related inconsistencies. As the volume of iEEG data continues to grow, there is a pressing need for automated methods to support accurate and scalable electrographic seizure classification (Ulate-Campos et al., 2016).

Traditionally, electrographic seizure classification approaches have relied on hand-engineered features extracted from EEG or iEEG signals using signal processing techniques such as the Fourier transform. The Fourier transform decomposes the signal into its constituent frequency components, enabling the identification of spectral patterns that may be indicative of seizure activity. These spectral features, such as power in specific frequency bands (for example, delta, theta, alpha, beta, gamma), are often used to characterize neural activity over time (Wang and Mengoni, 2022; Bandarabadi et al., 2014; Kharbouch et al., 2011; Netoff et al., 2009). Once extracted, these features are typically fed into conventional machine learning classifiers such as support vector machines, decision trees, k-nearest neighbors, or random forests. This pipeline of manual feature extraction followed by classification has shown reasonable performance in controlled settings (Netoff et al., 2009; Chan et al., 2008; He et al., 2022; Zhang and Parhi, 2015; Abbasi and Goldenholz, 2019). However, it is highly dependent on domain expertise and often requires extensive tuning of feature sets to perform well across different patients and seizure types. In addition, these hand-crafted approaches may fail to capture complex, non-linear relationships in the iEEG data and can struggle to generalize in real-world clinical scenarios, particularly when faced with inter-patient variability or noisy data (Kim et al., 2020; Xu et al., 2020).

Deep learning addresses many of the limitations inherent to traditional hand-crafted feature-based approaches. Unlike conventional methods that depend on expert-designed features and signal transformations, deep learning models learn hierarchical and task-specific representations directly from raw or minimally processed data. This ability to automatically discover relevant patterns enables models to capture complex temporal and spectral dependencies that may be difficult to encode manually. Deep learning approaches have shown promising results in seizure classification (Natu et al., 2022; Nafea and Ismail, 2022).

Convolutional neural networks (CNNs) have emerged as a widely used deep learning approach for seizure classification. CNNs use convolutional layers to capture spatial and temporal relationships in the signals and have been successfully applied to both time-series and time-frequency (image-based) representations, showing effectiveness in detecting subtle patterns in brain activity associated with seizures (Rashed-Al-Mahfuz et al., 2021; Barry et al., 2021; Jia et al., 2022; Desai et al., 2023).

More recently, transformer models have gained traction for electrographic seizure classification due to their ability to model long-range dependencies in EEG data. While CNNs are effective at capturing local patterns through convolutional kernels, they inherently struggle with capturing global context and long-term temporal relationships, which are often critical in identifying seizure dynamics that unfold over extended time windows. Transformer models address this limitation through self-attention mechanisms, which enable them to dynamically weigh and relate information across all time points in the input sequence. This capability enables better modeling of the complex and distributed patterns in neural data associated with seizures. As a result, transformer-based approaches have shown strong potential in EEG-based seizure classification tasks (Vaswani et al., 2017; Zhu et al., 2024; Hussein et al., 2022; Lih et al., 2023).

In this study, we present a vision transformer (ViT)-based approach for electrographic seizure classification using time-frequency spectrogram representations of intracranial EEG (iEEG) recordings, aiming to distinguish between recordings that contain seizure activity and those that do not. Recent studies have applied vision transformers to seizure detection and prediction, but these efforts remain limited in scope. For example, ViT models have been proposed for cross-subject seizure detection in scalp EEG (Feng et al., 2026). Hybrid convolutional neural network (CNN)-ViT fusion models have been proposed to combine local and global feature representations for seizure detection (Li et al., 2025), while others have introduced channel-selection mechanisms to identify the most informative scalp EEG electrodes for seizure prediction (Qi et al., 2024). Additional work has explored multi-channel ViT for spatio-temporal spectral feature learning to capture global spatial and long-range temporal dependencies for seizure detection (Hussein et al., 2022; Shi and Liu, 2024). While these methods report promising performance, they are evaluated almost exclusively on small, curated scalp EEG datasets such as CHB-MIT (22 pediatric patients, 198 seizure events only) or Kaggle (2-3 patients), which severely limits both the diversity of seizure events and the heterogeneity of clinical presentations. Moreover, these studies do not evaluate generalization to different independent patient populations or epilepsy syndromes, and in particular none have demonstrated robustness to out-of-distribution cohorts. In contrast, our work leverages a large intracranial EEG dataset collected from patients with drug-resistant focal epilepsy implanted with the NeuroPace^®^ RNS^®^ System that contains over 80,000 seizure events in the first training fold alone (Table 1). Specifically, we train our ViT model on data from 113 patients comprising 136,878 iEEG recordings. We perform clinical validation on a separate expert-labeled dataset of 3010 iEEG recordings obtained from 241 patients. This dataset spans diverse electrode locations, including mesial temporal (MTL), neocortical (NEO), and thalamic (THAL) leads, as well as multiple recording types such as long episodes, scheduled recordings, and magnet-triggered events. We benchmark performance against widely used image-based CNN architectures and also time-series-based architectures: ResNet-50, standard 2D CNN, time-series transformer and 1D CNN. We also evaluate the generalizability of our model on a separate iEEG dataset from patients with idiopathic generalized epilepsy (IGE), recorded using the NeuroPace^®^ RNS^®^ System. To better understand how the model reaches its predictions and to identify potential areas for improvement, we conduct explainability and error analyses. The explainability analysis aims to uncover which features of the input spectrograms the model relies on when classifying seizure and non-seizure events, providing insight into its decision-making process. Complementing this, the error analysis focuses on false positive and false negative predictions to highlight specific failure modes and guide future refinements in model design, data labeling, or training strategies. To our knowledge, this is the first ViT-based study to show such large-scale, clinically heterogeneous intracranial EEG evaluation with out-of-distribution generalization. Finally, while our present analysis focuses on single-channel classification to establish a clear benchmark, future work will extend these methods to multi-channel modeling to capture cross-channel interactions and further enhance performance. Ultimately, this work presents a ViT model for electrographic seizure classification, with the goal of assisting clinicians in reviewing large volumes of iEEG recordings from patients efficiently.

**Table 1:** Per-fold distribution of patients, seizures (Sz.), non-seizures (NSz.) and number of iEEG recordings across training, tuning, and test sets.

**Fold**	**Training set**				**Tuning set**				**Test set**			
	**Pts**.	**Sz**.	**NSz**.	# **recordings**	**Pts**.	**Sz**.	**NSz**.	# **recordings**	**Pts**.	**Sz**.	**NSz**.	# **recordings**
1	72	81,573	81,573	77,482	18	21,692	40,239	21,845	23	36,918	42,477	25,123
2	72	78,218	78,218	72,865	18	33,398	35,751	22,045	23	28,567	60,874	29,619
3	72	103,572	103,572	87,347	18	8,381	36,832	14,631	23	28,230	62,606	29,638
4	72	91,214	91,214	75,880	18	24,957	56,364	26,475	23	24,012	64,712	30,141
5	72	100,983	100,983	91,080	18	15,917	25,066	12,628	23	23,283	41,472	22,756

Sz. and NSz. show number of iEEG channels of each type.

## 2 Materials and methods

### 2.1 NeuroPace^®^ RNS^®^ System

**Figure 1:**
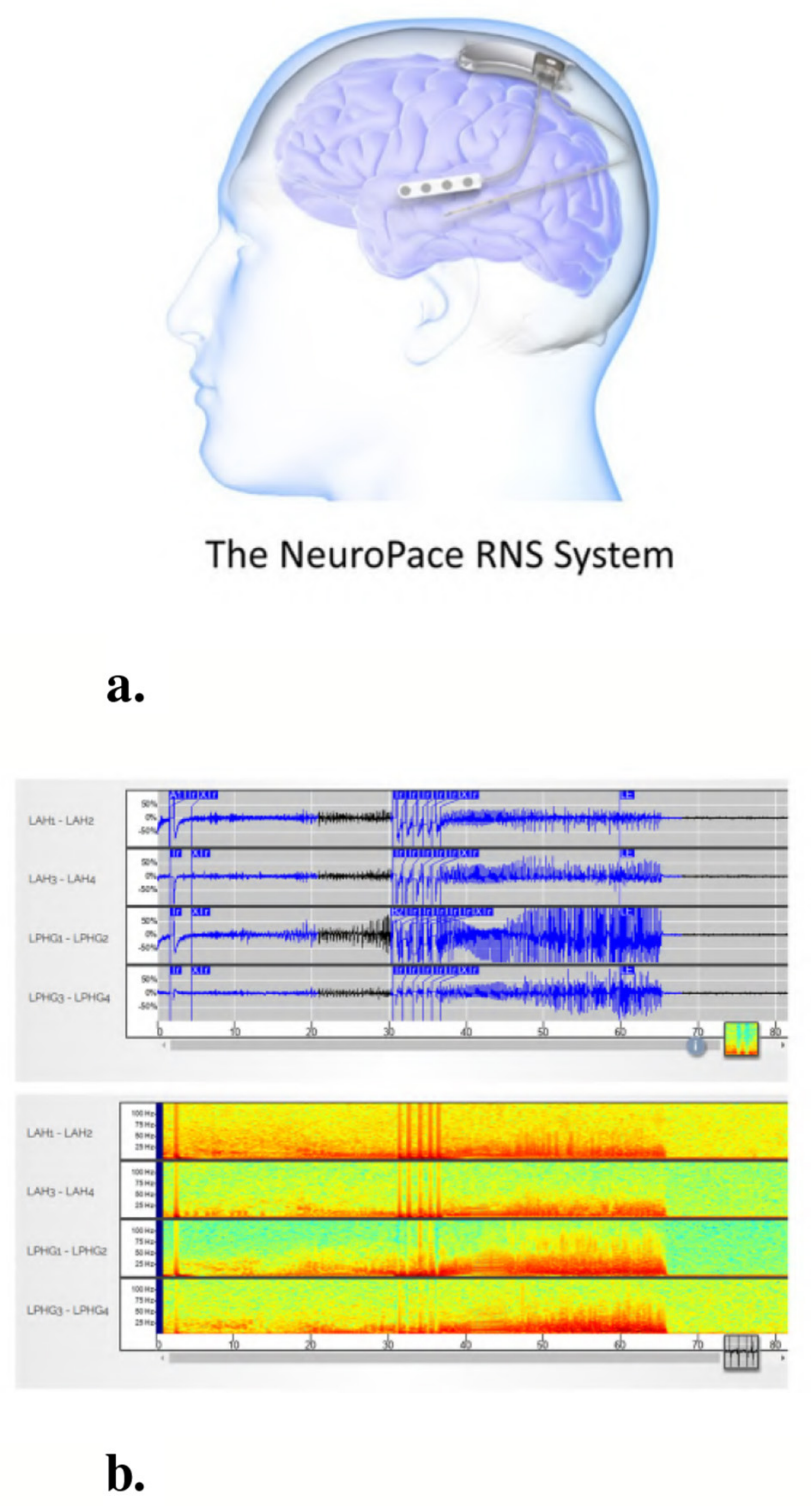
The NeuroPace^®^ RNS^®^ System and an example iEEG recording. **(a)** The NeuroPace^®^ RNS^®^ System includes an implanted neurostimulator connected to two leads, each containing four electrodes used for both sensing and stimulation. Leads are typically placed at the seizure onset zones. **(b)** Example 4-channel iEEG recording from an implanted device in a patient. The top panel shows raw time-series signals, and the bottom panel displays the corresponding time-frequency spectrograms for each channel.

The NeuroPace^®^ RNS^®^ System is an FDA-approved medical device for the treatment of patients with drug-resistant focal epilepsy involving one or two seizure foci. Detailed descriptions of the RNS^®^ System have been previously published (Bergey et al., 2015; Morrell, 2011; Skarpaas et al., 2019). The device operates as a closed-loop neurostimulation system, continuously monitoring intracranial brain activity and delivering electrical stimulation in response to detected abnormal patterns. Figure 1a illustrates the RNS^®^ System, while Figure 1b presents an example iEEG recording acquired from the device. Both the raw time-series signal and its corresponding time-frequency spectrogram representation are shown.

For the present study, we randomly selected data from 274 patients from the clinical trials of the RNS^®^ System for model training and evaluation. Feasibility, Pivotal and Long-Term Treatment (LTT) studies are registered on clinicaltrials.gov under identifiers NCT00079781, NCT00264810, and NCT00572195, respectively. Additionally, data from an independent cohort of 80 patients who were not enrolled in the clinical trials was also included for model evaluation. In total, data from 354 patients were analyzed in this study.

### 2.2 iEEG recording types

The RNS^®^ System captures several types of iEEG recordings, including long episode, scheduled, magnet and saturation recordings. Long episode recordings are initiated by the onboard detection algorithms in the device in response to prolonged abnormal brain activity. Scheduled recordings are typically collected twice daily and serve as baseline data for each patient. Magnet recordings are manually triggered by patients upon experiencing a clinical seizure. Saturation recordings are obtained when the device amplifiers become saturated.

In our dataset, long episode recordings constituted a large portion of the iEEG data and were found to contain both electrographic seizure activity and non-seizure activity across many patients. Therefore, we selected long episode recordings exclusively for model training. Each long episode iEEG recording consisted of 4 channels of time series signals sampled at 250 Hz. Although models were exclusively trained on long episode data, they were also evaluated on other categories of iEEG recordings, including scheduled, magnet, and saturation recordings.

### 2.3 Data splits

**Figure 2:**
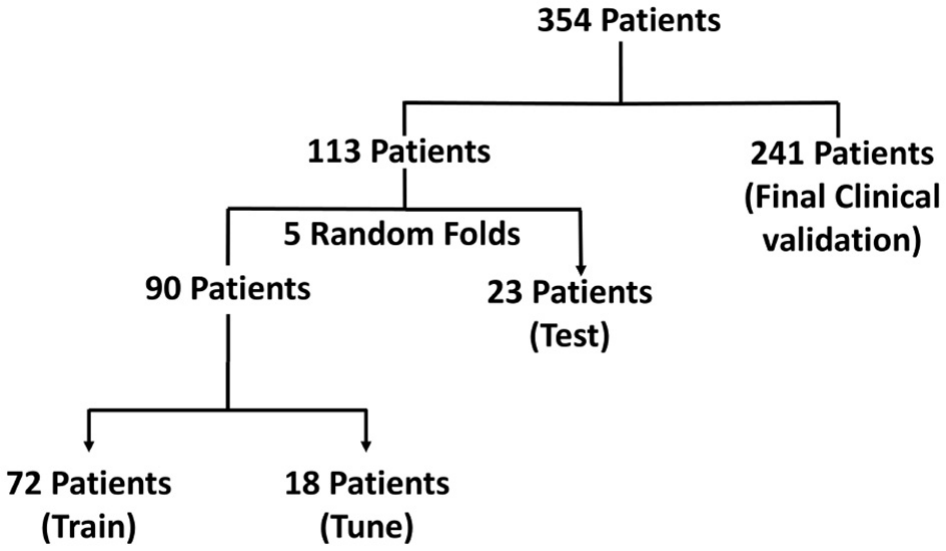
Visualization of the dataset used for model development and evaluation in the focal epilepsy population. The dataset includes iEEG recordings from 354 patients, with 113 patients used for training, tuning, and testing via 5-fold cross-validation, and 241 patients reserved for final clinical validation.

Of the 354 patients included, the recordings that formed part of the training set were obtained from 113 randomly selected patients with intracranial leads implanted in the mesiotemporal lobe or neocortex. 136,878 long episode iEEG recordings (approximately 547512 iEEG channels) from these 113 randomly selected patients were divided into five cross-validation folds, with each fold consisting of 72 patients for training, 18 for tuning, and 23 for testing. These 136,878 recordings were labeled by a trained NeuroPace^®^ employee. 3010 iEEG recordings (12040 iEEG channels) from 241 patients were used for clinical validation, with data labeled by three board-certified epileptologists. Among these, 161 patients had recordings from either mesiotemporal or neocortical leads, similar to the training set, and included long episode, scheduled, magnet, and saturation categories. The remaining 80 patients were implanted in the thalamus, and only their long episode recordings were used for clinical validation. The dataset splits are illustrated in Figure 2. Table 1 reports the total number of iEEG channels labeled as seizures and non-seizures, as well as the total number of iEEG recordings in the training, tuning, and test sets within the 5 folds of the 113-patient dataset. Table 2 summarizes the total number of recordings in the clinical validation dataset from 241 patients, along with the distribution of seizure and non-seizure recordings across the different iEEG categories (long episode, magnet, scheduled, and saturation).

**Table 2:** Total number of seizure and non-seizure iEEG recordings across the four categories (long episodes, magnet, saturation and scheduled) in the clinical validation dataset.

**iEEG category**	**Sz**.	**NSz**.	**Total number of recordings**
Long episodes	871	1,112	2,410
Magnet	30	146	200
Saturation	103	66	200
Scheduled	1	195	200

Sz. (seizure) and NSz. (non-seizure) counts reflect recordings with unanimous agreement among all expert reviewers, while the total number includes all recordings regardless of agreement level.

### 2.4 iEEG labeling process

The task of electrographic seizure classification involves distinguishing seizure activity from non-seizure baseline activity in iEEG recordings. To train and evaluate our AI models, we thus required supervised human-labeled iEEG data annotated as either seizure or non-seizure. A trained NeuroPace^®^ employee labeled the 113-patient dataset using a previously described clustering-based workflow (Barry et al., 2021). Briefly, we transformed the time-series activity from each iEEG channel into a time-frequency spectrogram image, and passed those images through a pretrained GoogLeNet Inception-V3 model to extract channel-level features. We created iEEG recording-level features after concatenating the features from the 4 channels for each iEEG recording. We then reduced the dimensionality of these recording-level features using PCA followed by t-SNE. A Bayesian Gaussian Mixture Model was then applied to cluster these recordings on a per-patient basis. The labeler manually assigned seizure and non-seizure labels to each cluster centroid and propagated these labels to all recordings within the corresponding clusters. To ensure labeling quality, the labeler visually reviewed all recordings in each cluster using a thumbnail-based interface and corrected any labels that did not align with the overall cluster characteristics. This approach yielded channel-level seizure and non-seizure labels for all iEEG recordings in the 113-patient dataset.

### 2.5 Model architecture

We used a vision transformer (ViT) architecture for the task of electrographic seizure classification. Specifically, we used the ViT-B/16 variant, which consists of 12 transformer layers, each with 12 self-attention heads, a hidden size of 768, and an MLP dimension of 3,072. A dropout rate of 0.1 was applied. The input data were split into 16 × 16 pixel patches. For our final model, all 86 million parameters in the ViT were fully trainable, and we call this model ViT (86M). To accommodate computational constraints in some experiments, we used a smaller variant of the model, referred to as ViT (49M), which contained 49 million trainable parameters. All references to the “ViT model” in this paper refer specifically to the ViT (86M) variant.

For the binary electrographic seizure classification task, we used the binary cross-entropy loss. We monitored validation accuracy during training with early stopping after 10 epochs of no improvement. Hyperparameters such as learning rate, optimizer, and dropout were selected through manual tuning based on preliminary experiments. The final model was trained with a learning rate of 1e-7, batch size of 32, and the Adam optimizer. The model produces a probability score ranging from 0 to 1, with higher values indicating a greater likelihood of seizure activity in the iEEG channel.

### 2.6 Data preprocessing

**Figure 3:**
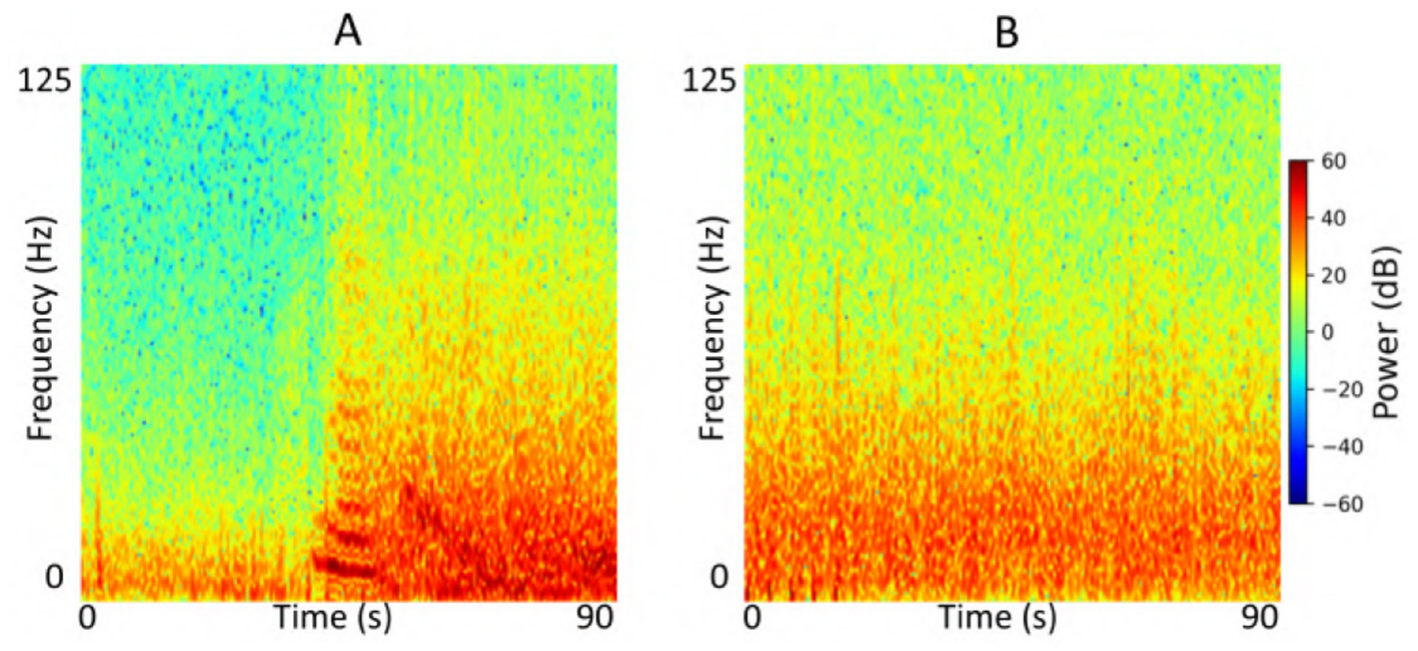
**(A)** Example iEEG channel spectrogram labeled as “seizure” by a human expert. **(B)** Example iEEG channel trace labeled as “non-seizure”.

**Figure 4:**
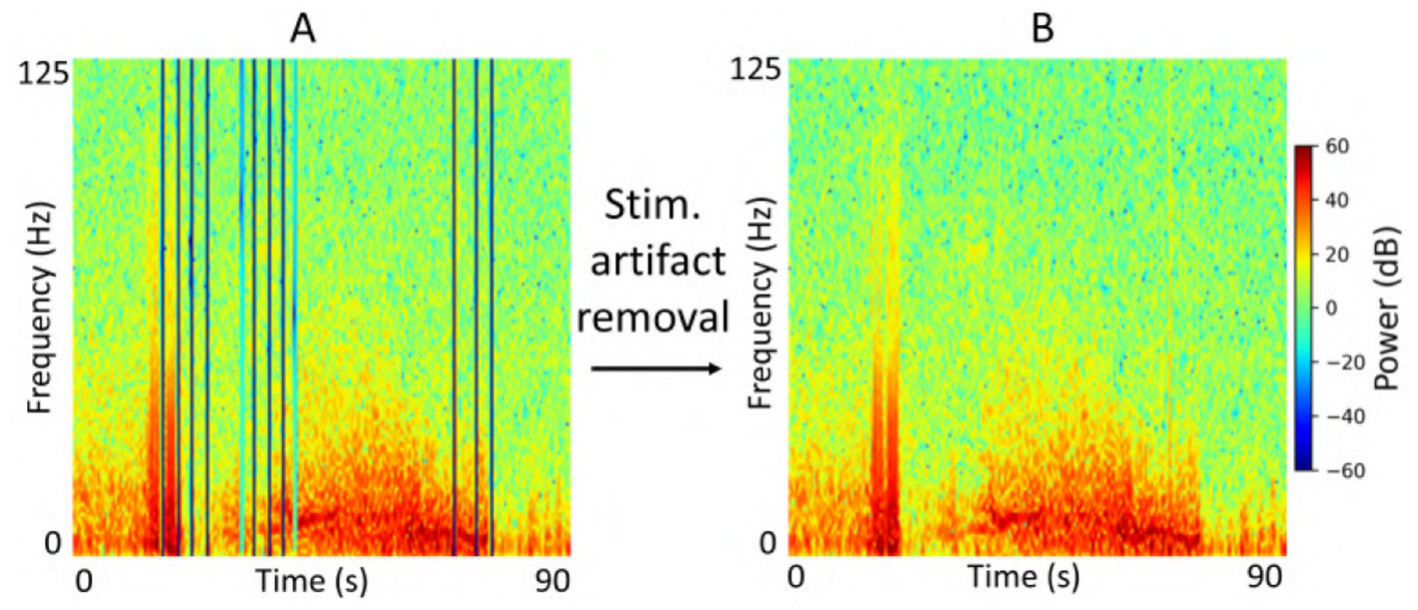
**(A)** Example iEEG channel spectrogram containing stimulation-related artifacts. **(B)** Spectrogram of the same iEEG channel after artifact removal.

Each iEEG recording included four channels of time-series activity, many of which contained neurostimulation artifacts. Since we had access to the precise onsets and durations of stimulation within each recording, we first removed the stimulation artifacts from all channels. We then converted the artifact-free signals into time-frequency spectrograms using the short-time Fourier transform implemented via the scipy.signal.spectrogram function. Signals were sampled at 250 Hz, with a window size of 128 samples, 80% overlap, and an FFT length of 512 points. We visualized the resulting spectrograms using a jet colormap and saved them as PNG images, which served as the input to the ViT model for electrographic seizure classification. Each image input was of size 224 × 224 × 3. Figure 3 shows examples of iEEG channels labeled as seizure and non-seizure activity. Similarly, Figure 4 shows a time-frequency spectrogram of an iEEG channel before and after stimulation artifact removal.

### 2.7 Experiments

**Figure 5:**
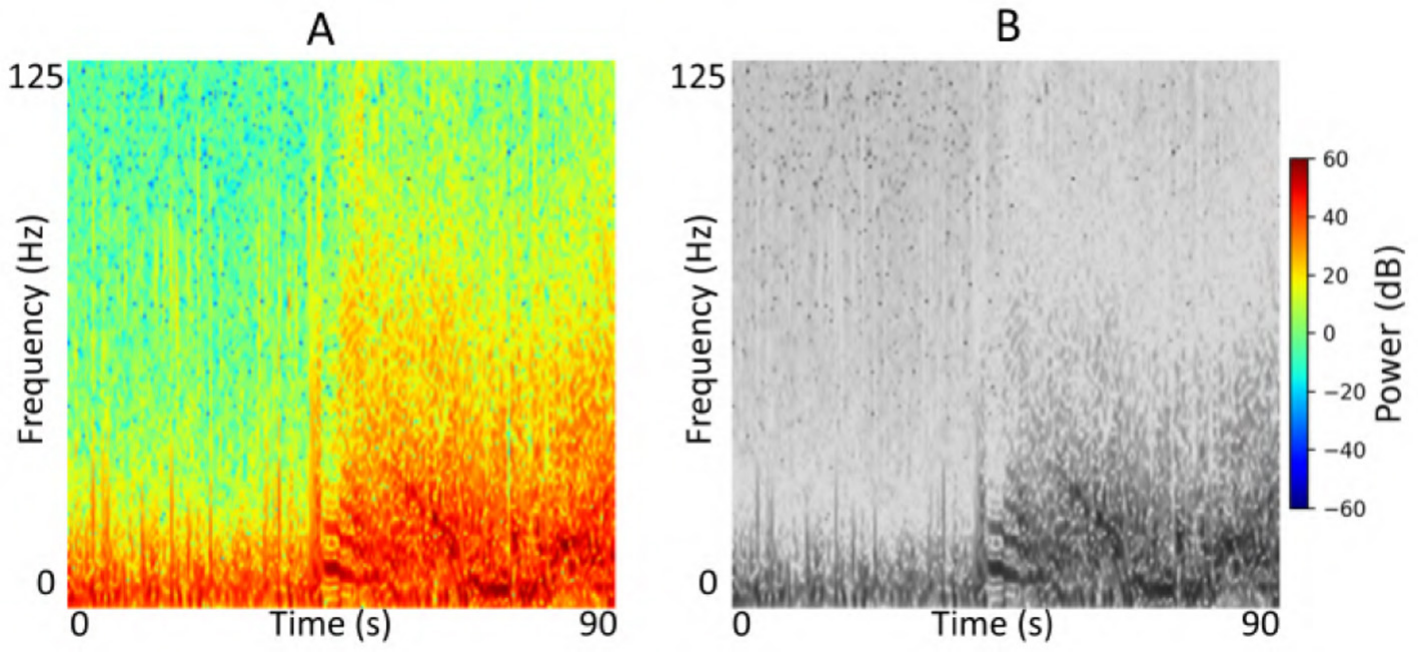
**(A)** Example color iEEG channel spectrogram visualized using the jet colormap. **(B)** Spectrogram of the same channel displayed in grayscale.

We conducted a series of experiments to evaluate the performance and generalizability of the ViT models on the task of electrographic seizure classification. First, we assessed performance within each cross-validation fold using 23-patient held-out test sets. In addition, we evaluated the model on the clinical validation dataset comprising 241 patients whose recordings were independently labeled by expert reviewers. To investigate the effect of stimulation artifact on model performance, we compared results using inputs with and without stimulation artifact removal. We also examined the impact of input format by comparing model performance on grayscale vs. color time-frequency spectrograms, specifically to assess the impact of using three RGB channels vs. a single-channel input. Figure 5 shows an example of an iEEG channel represented as both color and grayscale time-frequency spectrograms. Due to computational constraints, we used the ViT (49M) model for experiments assessing the impact of color information and stimulation artifact on model performance. Finally, we evaluated the model on a small, expert-labeled iEEG dataset from the IGE population (thalamic recordings) to assess seizure classification performance within this cohort. This iEEG dataset consisted of 136 recordings collected from 44 patients.

We additionally compared the ViT models against two commonly used image-based architectures: ResNet-50 and a 2D convolutional neural network (2D CNN). For the ResNet-50 model, we fine-tuned all layers of an ImageNet pretrained base model and extended it with a global average pooling layer followed by a softmax classification layer to form the final architecture. The model was trained using the Nadam optimizer with a learning rate of 1e-7 and a batch size of 32. Validation accuracy was monitored for early stopping and model checkpointing. ResNet-50 had approximately 23 million trainable parameters. The 2D CNN model consisted of three Conv2D layers with increasing filter sizes (32, 64, and 128), each using a 3 × 3 kernel with ReLU activation, followed by 2 × 2 max pooling. The extracted feature maps were flattened and passed through a dense layer with 256 units and ReLU activation, followed by a softmax output layer. This model was trained using the Adam optimizer with a learning rate of 1e-5 and a batch size of 32. It had approximately 22 million trainable parameters. We also included two time-series models for comparison, including a time-series transformer and a 1D CNN (Table 3). The time-series transformer used an initial Conv1D + pooling stack followed by 6 transformer encoder blocks (4 heads, head size 256) with residual connections and dropout, ending in global average pooling and a two-layer MLP classifier. The 1D CNN used a deep Conv1D architecture with 8 convolution + pooling layers of increasing filter sizes, followed by global average pooling and a dense classifier.

**Table 3:** Performance of the electrographic seizure classification models on the 23-patient test sets in each cross-validation fold.

**Model type**	**Image input**	**Fold 1**	**Fold 2**	**Fold 3**	**Fold 4**	**Fold 5**	**Mean** ±**SD**
ResNet-50	Color	96.2	95.4	95.3	95.8	95.5	95.6 ± 0.4
ResNet-50	Grayscale	95.4	94.0	94.5	94.6	95.2	94.7 ± 0.5
ViT (49M)	Color	96.6	96.1	94.9	96.7	95.7	96.0 ± 0.6
ViT (49M)	Grayscale	96.5	93.6	94.7	95.3	95.2	95.1 ± 0.9
**ViT (86M)**	**Color**	**96.9**	**96.5**	**96.0**	**97.2**	**97.1**	**96.8** ±**0.5**
2D CNN	Color	94.7	93.8	93.9	95.6	94.2	94.4 ± 0.6
2D CNN	Grayscale	94.9	94.2	94.2	94.5	92.6	94.1 ± 0.8
Time-series Transformer	n/a	93.4	87.3	91.0	95.9	90.8	91.7 ± 3.0
1D CNN	n/a	92.1	84.5	93.6	90.1	83.0	88.7 ± 4.2

The performance of the best-performing model is shown in bold.

### 2.8 Performance metrics

To evaluate the performance of all models, we used accuracy, which measures the overall proportion of correct predictions, and the F1 score, which balances precision and recall and is particularly useful in the presence of class imbalance.

## 3 Results

### 3.1 Model performance on test sets

We evaluated the performance of all models on the task of electrographic seizure classification. The models were evaluated on 23-patient test sets across 5 cross-validation folds, as shown in Figure 2. The models were evaluated on individual iEEG channel inputs from all recordings in the 23-patient dataset, with the task of determining whether each channel belonged to the seizure or non-seizure class. Unless otherwise specified, models in this analysis were evaluated after stimulation artifacts were removed from iEEG recordings as a preprocessing step. Table 3 shows binary classification accuracies for each of the five folds, and the final column presents the mean ± standard deviation (SD) of test accuracy across folds. The ViT (86M) model achieved the highest average test accuracy of 96.8%, making it the best-performing model in this comparison (color spectrogram inputs), outperforming other image-based and time-series-based models.

**Figure 6:**
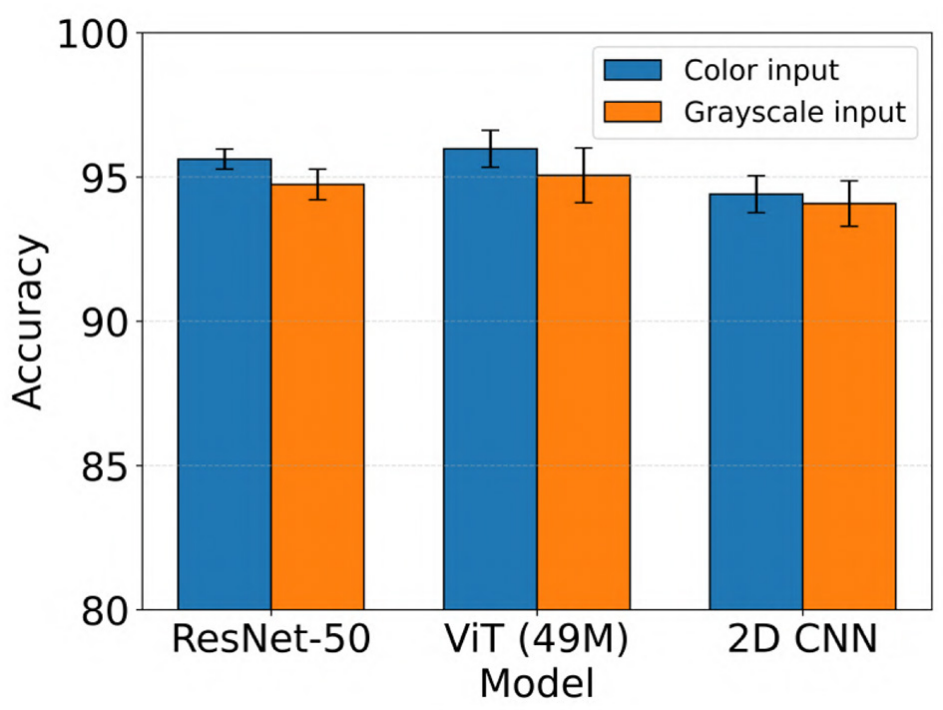
Performance comparison of electrographic seizure classification models using color vs. grayscale spectrogram inputs across ResNet-50, ViT (49M), and 2D CNN architectures. Error bars indicate the mean ± standard deviation of test accuracy across five cross-validation folds.

We evaluated the impact of using color vs. grayscale spectrogram inputs for our electrographic seizure classification models. Across models, performance was generally higher with color spectrogram images compared to their grayscale counterparts, as shown in Figure 6. For example, the ViT (49M) model achieved a mean test accuracy of 96.0% with color inputs, compared to 95.1% with grayscale inputs (this difference was not statistically significant). In contrast, ResNet-50 showed a statistically significant improvement with color inputs, achieving 95.6% accuracy vs. 94.7% with grayscale (Wilcoxon rank-sum test, Bonferroni-corrected *p* < 0.02). Overall, the use of color spectrograms provided a small but consistent performance advantage across models.

**Figure 7:**
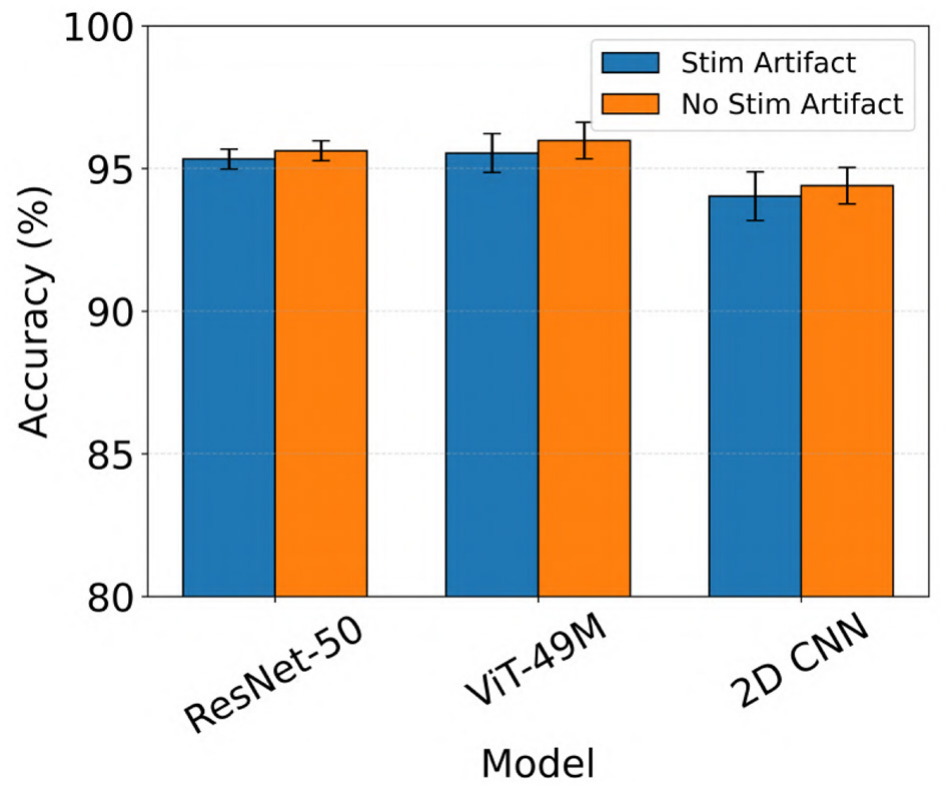
Performance comparison of electrographic seizure classification models before vs. after stimulation artifact removal across ResNet-50, ViT (49M), and 2D CNN architectures. Error bars indicate the mean ± standard deviation of test accuracy across five cross-validation folds.

Generally, all models performed slightly better when stimulation artifacts were removed from the iEEG data (not statistically significant, Wilcoxon rank-sum test). For example, ViT (49M) improved from a mean test accuracy of 95.5% to 96.0% when stimulation artifacts were removed (color inputs). The results are shown in Figure 7.

### 3.2 Model performance on clinical validation dataset

For the expert-labeled clinical validation dataset, we evaluated model performance at the iEEG recording level. The true label for a recording was considered seizure if any of its channels were labeled as a seizure by the expert, and non-seizure if all channels were labeled as non-seizure. Similarly, the predicted label for a recording was considered seizure if any of its channels were classified as seizure by the model, and non-seizure if all channels were predicted as non-seizure. For the ViT model, we identified an optimal operating threshold of 0.8 based on the tuning sets across the 5 cross-validation folds. This threshold yielded a high F1 score of 95.1% and a low false positive rate of only 0.8%. Consequently, we adopted 0.8 as the operating threshold for subsequent evaluation. Under this criterion, if any channel within a recording had a predicted probability greater than or equal to 0.8, the model classified the recording as a seizure. Conversely, if all channels had probabilities below 0.8, the recording was classified as non-seizure.

We evaluated the performance of all models against the three expert labelers in classifying recordings from the 241-patient clinical validation dataset. The results of this comparison are presented in Table 4. The table reports binary seizure classification accuracy and F1 scores for the best-performing models from the 23-patient test sets. Model comparisons against each expert labeler are shown in separate columns (*exp*. denotes expert). On this dataset, we observed notable performance differences across models, reflected in both accuracy and F1 scores. Among all models, ViT (86M) consistently outperformed others, achieving accuracy scores of 90.2%, 91.3%, and 87.5% when compared to expert 1, expert 2, and expert 3, respectively.

**Table 4:** Performance of the electrographic seizure classification models on the clinical validation dataset.

**Model**	**Accuracy**			**F1 score**		
	**vs. Exp. 1**	**vs. Exp. 2**	**vs. Exp. 3**	**vs. Exp. 1**	**vs. Exp. 2**	**vs. Exp. 3**
ResNet-50	86.9	87.5	85.8	84.3	84.7	84.7
**ViT (86M)**	**90.2**	**91.3**	**87.5**	**87.5**	**88.6**	**85.8**
2D CNN	83.0	83.5	83.1	80.7	80.9	82.6

The performance of the best-performing model is shown in bold.

**Figure 8:**
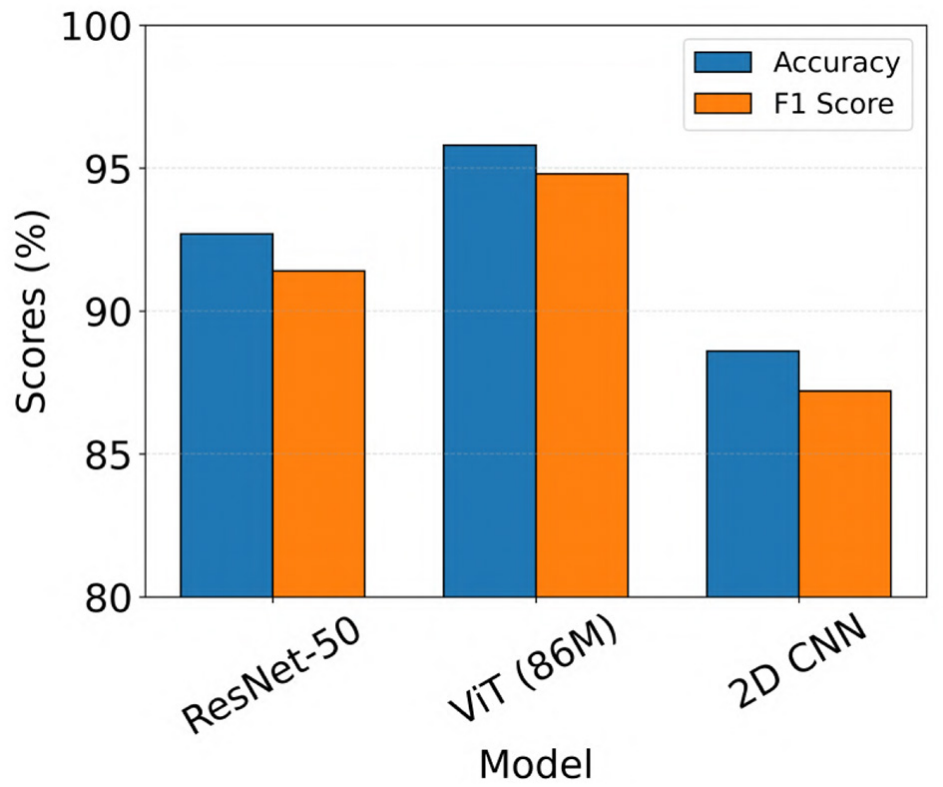
Model performance on iEEG recordings from the clinical validation dataset with unanimous expert agreement on seizure and non-seizure labels.

Of the 3010 iEEG recordings labeled by clinical experts for final model evaluation, 2524 recordings showed full agreement among all three experts, with unanimous labels as either seizure or non-seizure. For subsequent analysis, we assessed the performance of the top-performing ViT, ResNet-50, and 2D CNN models, which were identified from the 23-patient test set, on this subset of unanimously labeled recordings. Accuracy and F1 scores for these recordings are presented in Figure 8. As shown in the figure, ViT (86M) showed the highest performance, achieving a seizure classification accuracy of 95.8% and an F1 score of 94.8%, outperforming both ResNet-50 and 2D CNN models. We performed formal statistical comparisons of the three models (ViT (86M), ResNet-50 and 2D CNN) on these 2524 iEEG recordings in the clinical validation dataset. Specifically, we first applied Cochran's Q test across all three models, which revealed a highly significant global difference (*Q* = 147.72, *p* = 8.36 × 10^−33^). To identify pairwise differences, we then conducted McNemar's tests with Holm correction for multiple comparisons. These analyses showed that ViT (86M) significantly outperformed both ResNet-50 (χ^2^ = 36.33, *p* < 1.7 × 10^−9^) and 2D CNN (χ^2^ = 122.24, *p* < 6.2 × 10^−28^).

The patients in the clinical validation dataset had leads implanted in several locations, including the mesial temporal lobe (MTL), thalamus (THAL), neocortex (NEO), and cases with leads in both the mesial temporal lobe and neocortex (MTL-NEO). To further evaluate the performance of ViT (86M), we analyzed the 2524 recordings with full expert agreement and calculated accuracy and F1 scores stratified by lead location. These results are summarized in Table 5. The table shows that the model performs robustly across all lead locations, achieving greater than 90% accuracy in each lead location category.

**Table 5:** Accuracy and F1 scores of the ViT (86M) model by lead location, on a subset of the clinical validation dataset from 241 patients where all experts agreed on seizure vs. non-seizure labels.

**Lead Location**	**Accuracy**	**F1 Score**
MTL	97.4	97.3
MTL-NEO	95.5	94.3
NEO	92.4	90.1
THAL	98.1	96.8

**Figure 9:**
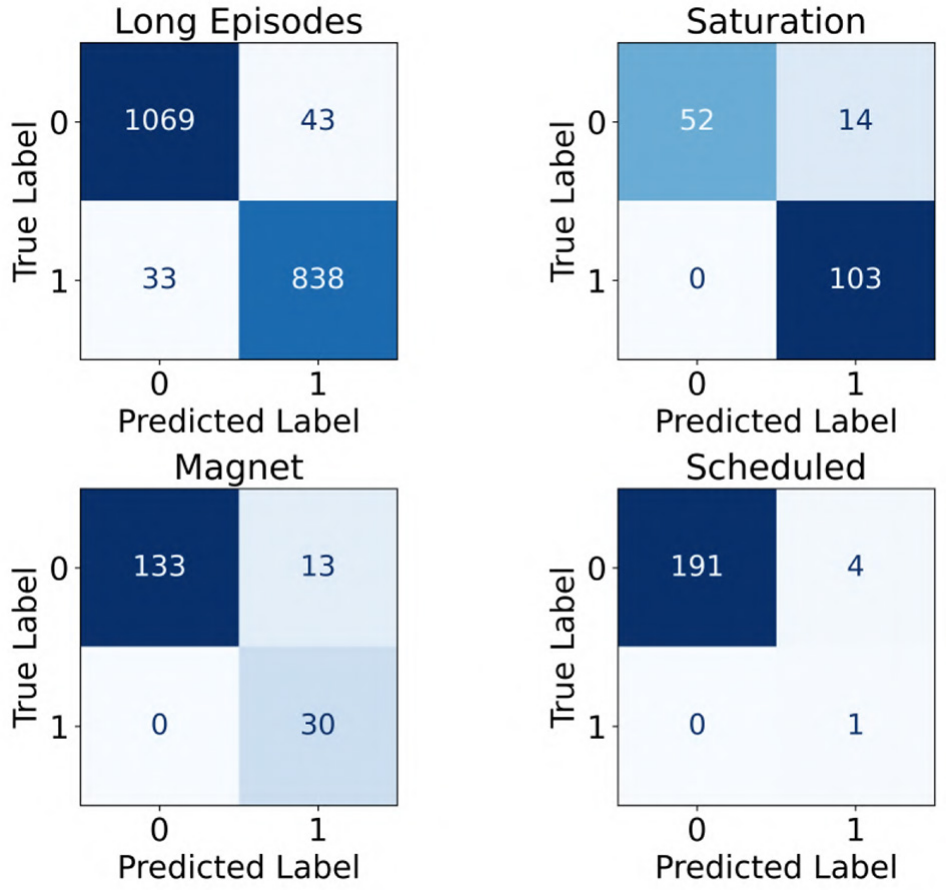
Confusion matrices showing the performance of the ViT model across four categories of iEEG recordings: long episodes, saturation, magnet, and scheduled. A label of “1” indicates seizure class, whereas “0” denotes non-seizure class.

Further, since the iEEG recordings included four distinct categories (long episodes, scheduled, magnet, and saturation recordings) with varying characteristics, we evaluated the performance of the ViT (86M) model separately across these categories. Notably, the model was trained exclusively on long episode recordings. The results, shown as confusion matrices in Figure 9, reflect performance on the subset of recordings where all three experts provided unanimous labels in the clinical validation dataset (241 patients). Across all categories, the model showed strong performance, with high counts of true positives and true negatives. Three out of the four categories exhibited no false negatives, and false positive rates remained low throughout, suggesting good generalizability of the model across diverse iEEG event types.

**Figure 10:**
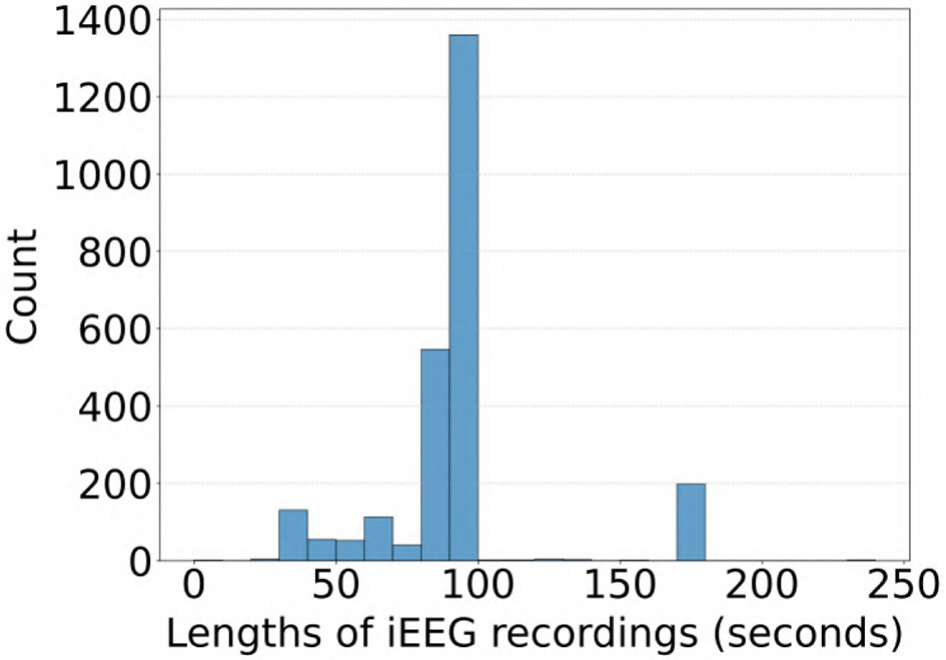
Distribution of iEEG recording durations in the subset of the clinical validation dataset with full expert agreement on labels.

Most of the iEEG recordings in our dataset were approximately 90 seconds in duration sampled at 250 Hz, with some recordings extending to 180 seconds and others less than 60 seconds. A distribution of durations for the 2,524 recordings in the clinical validation dataset where all experts agreed on labels is shown in Figure 10. To evaluate whether recording duration influenced model performance, we analyzed ViT (86M) accuracy on the subset of the clinical validation dataset with full expert agreement, stratified by recording duration (< 50s, >50- ≤ 100s, and >100s). As shown in Table 6, accuracies were comparable across these three categories, indicating that recording duration did not affect model performance.

**Table 6:** ViT (86M) model accuracy on iEEG recordings stratified by recording duration in the clinical validation dataset with full expert agreement.

**iEEG recording duration**	**Number of recordings**	**Accuracy**
< 50s	194	93.2
>50– ≤ 100s	2,117	96.1
>100s	213	95.7

### 3.3 Generalization to an idiopathic generalized epilepsy (IGE) dataset

**Figure 11:**
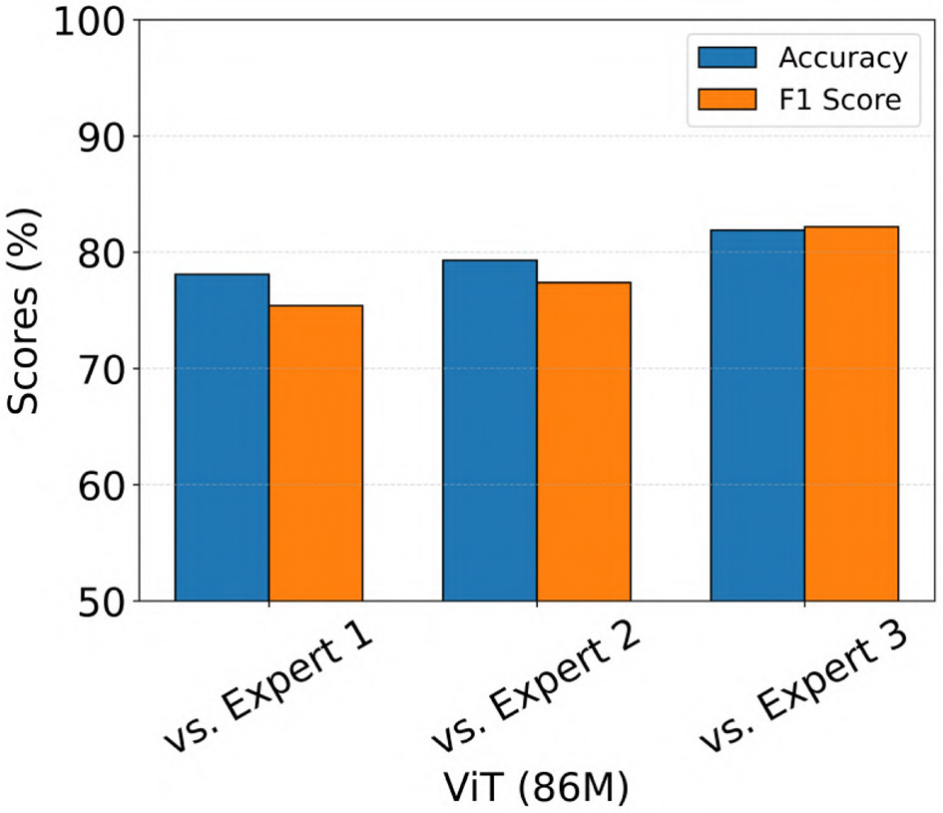
Performance of the ViT model on an out-of-distribution dataset consisting of iEEG recordings from patients with IGE implanted in the thalamus with the NeuroPace^®^ RNS^®^ system.

We also evaluated the performance of the ViT (86M) model on a separate iEEG dataset consisting of 136 recordings from 44 patients with IGE who were implanted in the thalamus with the NeuroPace^®^ RNS^®^ System. The electrographic characteristics in the IGE population may differ from those in the focal epilepsy population (Seneviratne et al., 2011), on which the model was originally trained. Therefore, this evaluation aimed to assess the ability of the model to generalize to recordings from the IGE population. The same 3 epileptologists independently labeled each iEEG recording as a seizure if any channel exhibited generalized tonic-clonic (GTC) seizure activity, a primary seizure type in IGE. Recordings were labeled as non-seizure if no channels showed evidence of GTC activity. The model predictions were compared against expert labels, and the results are shown in Figure 11. The model achieved accuracy and F1 scores exceeding 70% across all three comparisons, with the highest performance observed against Expert 3, yielding both accuracy and F1 score values of 82%.

### 3.4 Explainability

**Figure 12:**
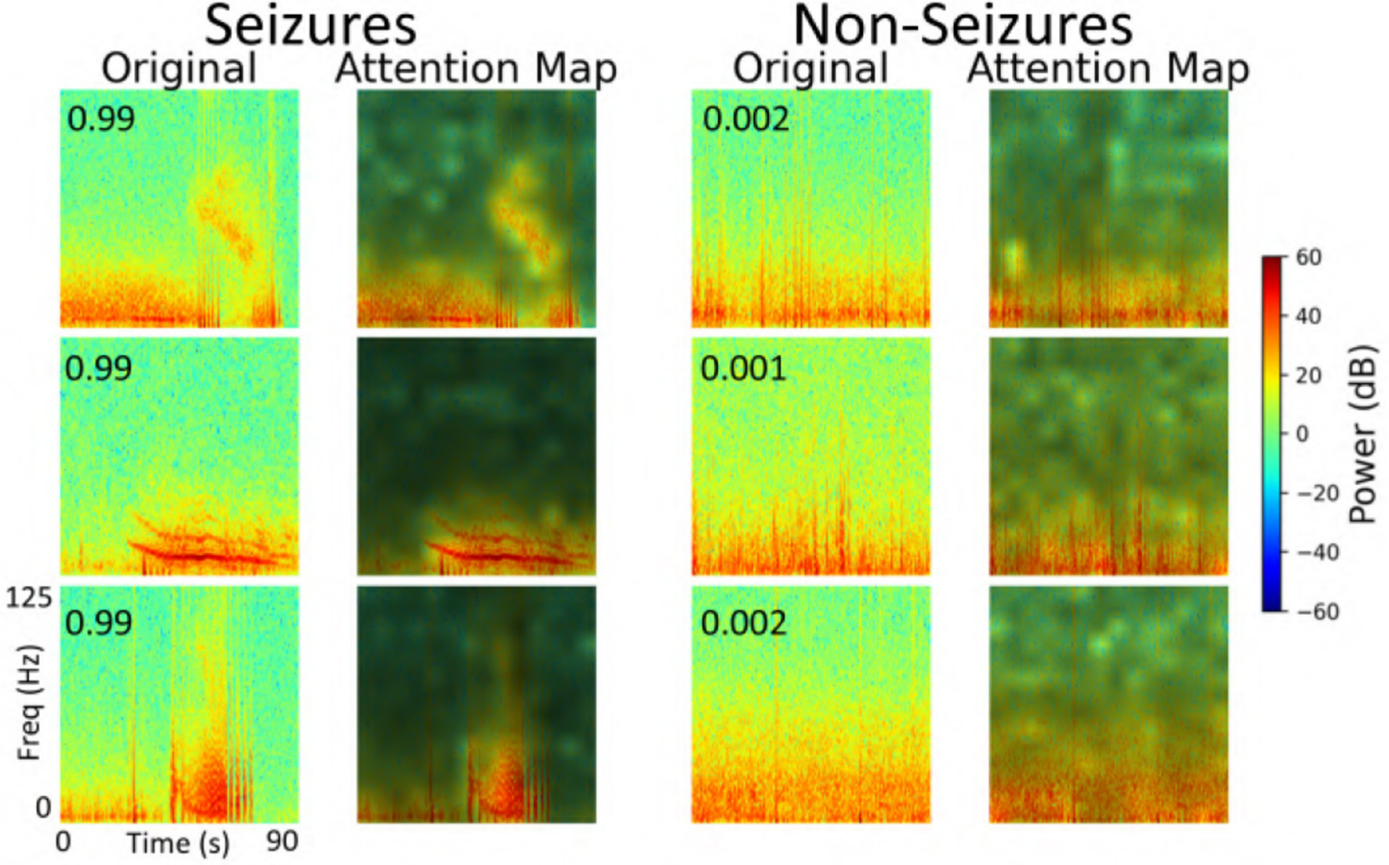
Explainability analysis showing attention maps for three high-confidence seizure and non-seizure examples. For each class, each row displays an iEEG spectrogram **(left)** alongside its corresponding attention map **(right)**, highlighting the regions the model focused on during classification. The number inside the **left panel** indicates the seizure probability predicted by the model.

We performed an explainability analysis to investigate which regions of the input spectrograms influenced seizure and non-seizure classifications by the ViT model. To do this, we applied an attention rollout technique (Abnar and Zuidema, 2020), which aggregates attention weights across all transformer layers to identify the input regions that most contributed to the final classification. Attention maps were computed for iEEG spectrogram channels in the test set of a representative fold from the 5-fold cross-validation. From these, we randomly selected 3 predictions where the model indicated high confidence in the seizure class, and 3 where high confidence was assigned to the non-seizure class. The resulting attention maps are shown in Figure 12. In these maps, brighter regions indicate areas that contributed more strongly to model decisions, reflecting where attention was most concentrated during classification. In the 3 example predictions where the model assigned high probability to the seizure class, the corresponding attention maps show concentrated focus on high-frequency bands in the iEEG channel spectrograms. These regions are consistent with electrographic seizure activity typically associated with focal seizures. In contrast, the 3 non-seizure examples exhibit more diffuse attention patterns spread across the entire spectrograms, with no dominant focus on any specific region. This scattered attention reflects the absence of characteristic seizure features, resulting in a low predicted probability for seizure class.

### 3.5 Error analysis

We conducted an error analysis across all 5 cross-validation folds to evaluate ViT model performance on the 113-patient dataset. Specifically, we quantified the percentage of false positives and false negatives in the test sets of each fold using an operating threshold of 0.8 for the predicted seizure probabilities. On average, the ViT model yielded 0.9% ± 0.2% false positives and 8.7% ± 2.9% false negatives across the 5 folds. False negatives were defined as seizure-labeled iEEG channels in the test set that were misclassified by the model as non-seizure, and false positives were non-seizure labeled channels misclassified as seizures.

**Figure 13:**
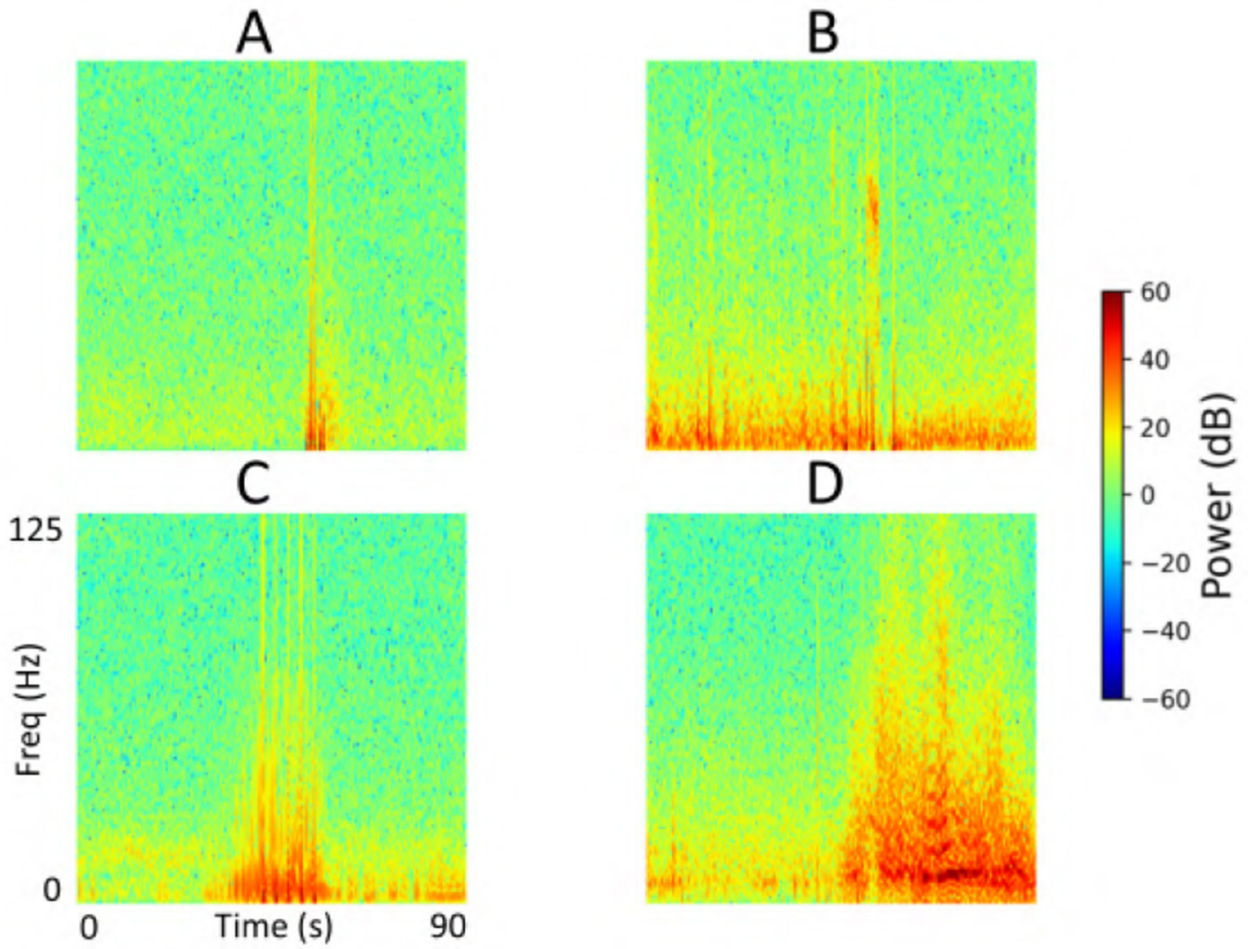
Cluster centroids from iEEG channels identified as false positives by the model in the test set of a representative fold from the 5-fold cross-validation. **(A, B)** Show cluster centroids that were correctly labeled as non-seizure by the human labeler, as they lack sustained or evolving ictal activity, but were incorrectly classified as seizures by the model. In contrast, **(C, D)** display clusters that likely reflect true seizure activity, suggesting the model predictions may have been accurate and the original human labels could be reevaluated.

**Figure 14:**
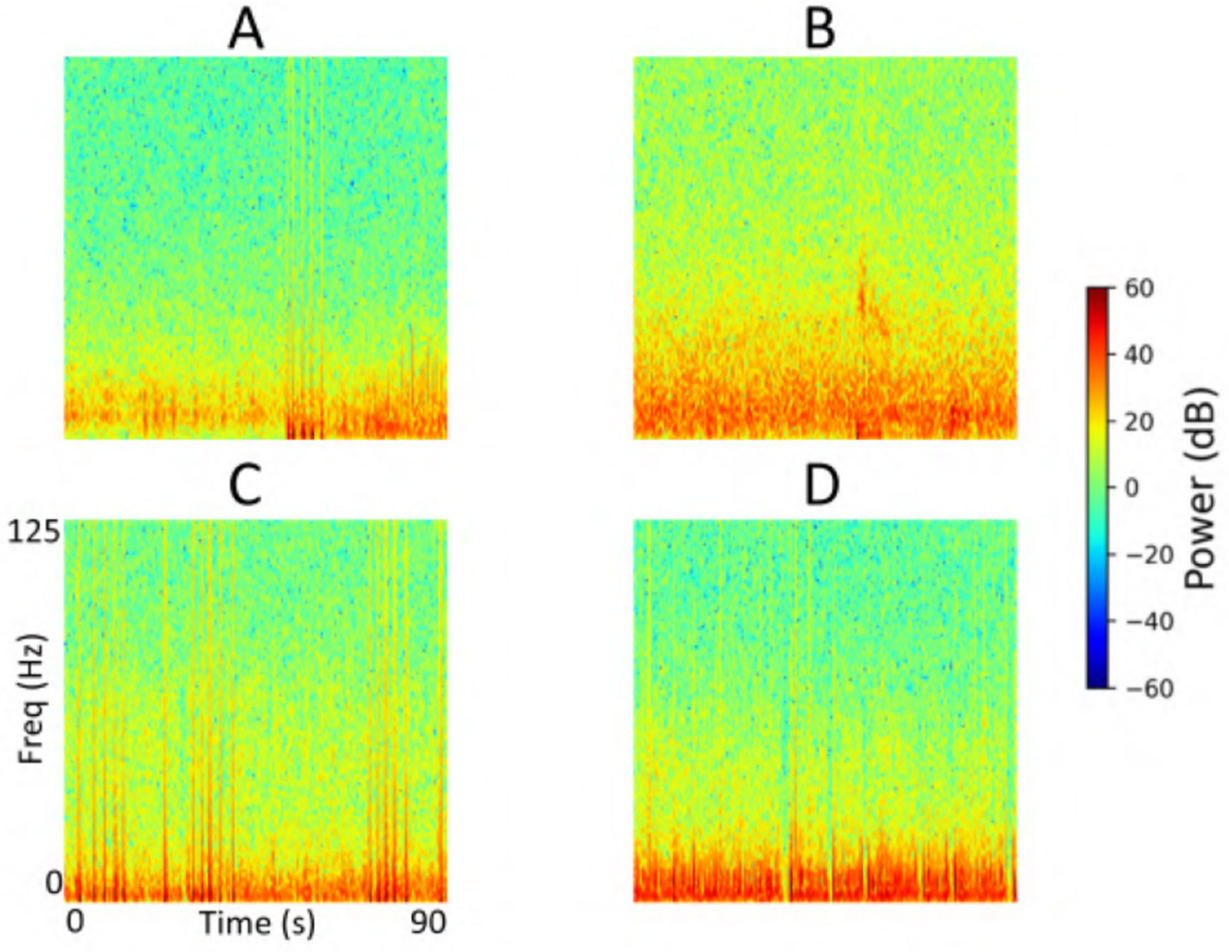
Cluster centroids from iEEG channels identified as false negatives by the model in the test set of a representative fold from the 5-fold cross-validation. **(A, B)** Display cluster centroids exhibiting some seizure-like characteristics that were correctly labeled as seizures by the human labeler but were missed by the model. In contrast, **(C, D)** contain non-ictal patterns that the model correctly classified as non-seizures, suggesting these instances may not represent actual false negatives and that the original labels may need reconsideration.

A detailed breakdown of false positive and false negative percentages per fold is presented in Table 7. To further examine model errors, we selected one representative fold and applied the clustering methodology described in subsection 2.4 to cluster the iEEG channels corresponding to false positive and false negative predictions separately. Manual inspection of the resulting clusters revealed that several false positive clusters contained ambiguous signals that may have been justifiably predicted as seizures by the ViT model, and vice versa for false negative clusters. Representative cluster centroids for false positives and false negatives groups are shown in Figure 13, Figure 14, respectively. For false positives, in Figure 13, panels A and B show cluster centroids that were correctly labeled as non-seizure by human labeler, as they lack sustained or evolving ictal activity, yet were incorrectly classified as seizures by the model. Conversely, panels C and D depict clusters that likely reflect true seizure activity, suggesting that the model predictions may have been correct and the original human labels could be reconsidered. Similarly, in the false negative clusters shown in Figure 14, panels A and B display examples that show some seizure characteristics and were appropriately labeled as seizures by human labeler, but missed by the model. Panels C and D, however, contain non-ictal patterns that the model correctly identified as non-seizures, indicating that these instances may not represent actual false negatives.

**Table 7:** Percentage of false positive and false negative iEEG channels in the test sets of the 5 cross-validation folds.

**Fold number**	**False positive *%***	**False negative *%***
1	0.8	6.8
2	0.8	10.2
3	0.7	13.7
4	1.3	5.5
5	0.7	7.2

## 4 Discussion and conclusion

In this study, we applied a ViT model for the task of electrographic seizure classification using a large-scale iEEG dataset collected from patients implanted with the NeuroPace^®^ RNS^®^ System. The model was trained and evaluated using 5-fold cross-validation on data from 113 patients (136,878 iEEG recordings). ViT achieved an average test accuracy of 96.8% across the five folds. We further validated the model on a clinical validation dataset consisting of 3010 iEEG recordings from 241 patients. This dataset was annotated by 3 board-certified epileptologists. On the subset of recordings where all experts unanimously agreed on the seizure or non-seizure labels, the ViT model achieved an accuracy of 95.8% and an F1 score of 94.8% (Figure 8). This was followed by the ResNet-50 model with accuracy and F1 scores of 92.7% and 91.4%, respectively.

Notably, the ViT model consistently outperformed conventional image-based deep learning methods, including a 2D CNN and a ResNet-50 architecture. The superior performance of ViT compared to CNNs may be attributed to its ability to model long-range dependencies and global contextual information within the spectrogram inputs. Unlike CNNs, which rely on localized receptive fields and hierarchical feature extraction, the self-attention mechanism in ViT allows the model to attend to patterns across the entire input image simultaneously. This global perspective may be particularly advantageous in identifying electrographic seizure related features that manifest across broader temporal or frequency regions in the spectrograms.

In our work, we used time-frequency images as inputs to the vision transformer model. This choice allowed us to leverage pretraining on ImageNet, where the model has already learned useful representations of visual patterns such as edges, textures, and shapes. By starting from a pretrained state, we leveraged both the prior knowledge encoded during large-scale pretraining and the new knowledge acquired from supervised training on the seizure classification dataset. This approach yielded high classification accuracy, supporting the effectiveness of using image-based representations. A practical advantage of the image-based approach is the ability to tap into the rich ecosystem of pretrained models, toolkits, and optimization strategies developed in the computer vision community, which would not directly apply to one-dimensional raw spectral inputs. Moreover, we note that some prior studies in EEG seizure prediction have successfully adopted time-frequency image representations, further validating the suitability of this approach (Hussein et al., 2022; Barry et al., 2021).

The 136,878 recordings in the 113-patient training dataset were labeled by a NeuroPace employee who was not a board-certified epileptologist but was trained and supervised by three senior NeuroPace colleagues, including one board-certified epileptologist. To manage the large volume of data, labeling was aided by a clustering method described in subsection 2.4. In this process, the labeler manually assigned seizure vs. non-seizure labels to each cluster centroid, which were then propagated to all recordings in that cluster. To ensure quality, the labeler visually reviewed all recordings within each cluster using a thumbnail-based interface and corrected any mislabels that did not align with the cluster characteristics. The labeler also consulted with the board-certified epileptologist whenever uncertain about specific cases. This strategy enabled efficient generation of channel-level seizure and non-seizure labels for the 113-patient dataset. Because labeling such a large dataset is extremely time-consuming, board-certified epileptologists could not feasibly label all recordings directly. For the final clinical validation, therefore, we used a separate dataset of 3010 recordings from 241 patients, which was fully labeled by three board-certified epileptologists without clustering assistance.

We formed class-balanced iEEG training datasets across the 5 cross-validation folds ensuring that both seizure and non-seizure classes were equally represented. This made sure that the model could properly learn the discriminative characteristics of both classes rather than being biased toward the more prevalent class. In contrast, our tuning, test, and clinical validation datasets were not class-balanced, reflecting the natural distribution of seizure and non-seizure events as captured by the device in real-world settings. Table 2 shows the natural distribution of seizure/non-seizure examples by iEEG recording category in the clinical validation dataset. To account for this class imbalance, we reported F1 scores alongside accuracy in the evaluation of our models. The F1 metric, which balances precision and recall, provides a more informative assessment of model performance when class distributions are skewed.

The number of patients included in the training and clinical validation datasets was determined by practical limitations in human labeling capacity rather than a specific design choice. For the training set, 136,878 recordings from 113 randomly selected patients were labeled by a NeuroPace employee under the supervision of three employees, including a board-certified epileptologist, with a clustering tool used to accelerate the process (Barry et al., 2021). In contrast, the clinical validation set comprised 3010 recordings from 241 patients that were labeled independently by three board-certified epileptologists. This effort required several months and was conducted without any clustering support, making it especially time-consuming but also highly valuable as a source of expert-annotated ground truth.

Our dataset included various categories of iEEG recordings, namely long episodes, magnet swipes, scheduled recordings, and saturation events, as defined in subsection 2.2. For the cross-validation phase, we used long episodes exclusively from 113 patients. This decision was based on both the prevalence and content of long episodes: they represent approximately 50% of the full NeuroPace^®^ iEEG dataset and typically contain a mixture of both electrographic seizure activity and non-seizure baseline activity. As such, they provided a rich source of diverse examples for model training. During final clinical validation, we also assessed model performance across the different iEEG recording categories, as shown in Figure 9. The model showed strong performance across all categories, with low rates of both false positives and false negatives. These results suggest that although the model was trained exclusively on long episode recordings, it was able to generalize effectively to other types of recordings, including magnet, scheduled, and saturation events. This generalization may be attributed to the diversity within long episodes, which likely captured a wide range of electrographic patterns present across different recording types.

Although the model was primarily trained and evaluated on iEEG recordings from patients with focal epilepsy (mesiotemporal, neocortical or thalamic leads), we also tested its performance on a separate dataset consisting of 136 recordings from 44 patients with IGE (thalamic leads). In this dataset, expert reviewers labeled each recording based on the presence or absence of GTC seizure activity. The ViT model generalized to this epilepsy population, achieving accuracy and F1 scores exceeding 75% across all expert comparisons (Figure 11). These results suggest that the model is capable of extending beyond its training distribution and performing effectively in other epilepsy subtypes. One possible explanation is that GTC seizures may share certain time and frequency domain features with focal electrographic seizures, which the model was able to learn during training.

We found that using color time-frequency spectrogram inputs consistently improved model performance across all architectures (Figure 6). For both the ViT and ResNet models, ImageNet-pretrained weights were used to initialize the networks prior to training on our iEEG spectrogram dataset. It is possible that the color-specific representations learned during ImageNet pretraining contributed to better feature extraction when applied to color spectrograms. This transfer of color-relevant features may have enhanced the ability of the models to differentiate seizure from non-seizure activity in the iEEG datasets.

ViT showed slightly improved performance when stimulation artifacts were removed from the iEEG recordings, although the difference was not statistically significant (Figure 7). This modest improvement suggests that the ViT was relatively robust to the presence of artifacts and noise. Despite the added signal contamination, the model was still able to effectively learn the distinction between seizure and non-seizure activity, indicating resilience in real-world clinical scenarios where artifacts are often unavoidable.

For the 23-patient test datasets, we evaluated model performance at the iEEG channel level within each of the 5 cross-validation folds, assessing the classification of individual channels as seizure or non-seizure. In contrast, for the final clinical validation datasets, we conducted evaluation at the iEEG record level. Specifically, a recording was labeled as a seizure if any channel within it was marked as a seizure by the model or expert, and as a non-seizure only if all channels were labeled as non-seizure. This shift in methodology reflects real-world clinical priorities, where clinicians are primarily interested in identifying whether a seizure occurred in a recording regardless of the specific channel when reviewing large volumes of data over time. Given the scale of the datasets and the clinical relevance of recording-level trends, evaluating model performance at the record level provides a more meaningful measure of its utility in practice.

The explainability analysis (subsection 3.4) provides insight into how the ViT model processes iEEG spectrogram inputs when classifying seizure and non-seizure events. In high-confidence seizure predictions, attention was focused on localized high-frequency components of the spectrograms, which correspond well to electrographic features typically observed in focal seizures. This suggests that the model has learned to associate certain time-frequency patterns with seizure activity. In contrast, attention during non-seizure classifications was more diffusely distributed across the spectrograms, consistent with the absence of well-defined seizure signatures. This dichotomy between focused and scattered attention patterns adds interpretability to the model and offers a qualitative explanation for its outputs. While these findings are encouraging, several opportunities exist for improvements to this analysis. These maps could be reviewed by expert epileptologists to assess whether the model attends to meaningful electrographic features, enabling human-in-the-loop validation. Similarly, attention maps across incorrect predictions (false positives and false negatives) could be studied to identify systematic biases or failure modes in the model, potentially guiding targeted improvements in data labeling, model architecture, or training. In the example predictions shown in Figure 12, the high frequency bands contributed prominently to the classification of electrographic seizures by the model. Our focal dataset includes a wide range of electrographic seizure onset patterns, such as low-voltage fast activity, hypersynchronous onset, delta/theta onset, and semi-rhythmic spiking. The model may focus on different regions in the images depending on the onset pattern. A comprehensive characterization of onset patterns across the full dataset would require a dedicated labeling effort that is currently outside the scope of this work. Nonetheless, this is an important and interesting direction. In future work, we aim to develop a model that can classify between distinct seizure onset types using supervised labels of onset patterns, where similar explainability analyses could be performed to systematically examine how the model differentiates among distinct onset types.

The error analysis presented in subsection 3.5 revealed that while many false positive and false negative cluster centroids suggested opportunities for improved model performance through additional labeled data, there were also instances where the ViT model predictions appeared more accurate than the original human labels. This discrepancy may, in part, be attributed to the clustering methodology (described in subsection 2.4) used to assist the manual labeling process. While clustering facilitated scalable labeling, it may have also introduced inconsistencies or label noise in some cases. These findings highlight the importance of label quality in training and evaluating our AI models. Addressing these potential labeling errors by refining the clustering-assisted labeling workflow could enhance the reliability of the training data and improve model accuracy. Nonetheless, it is important to note that the overall percentage of false positives and false negatives across all folds was low, as summarized in Table 7, underscoring the robustness of the ViT model even in the presence of minor labeling inconsistencies. False positive percentages across all folds were consistently lower than false negative percentages, which is a favorable outcome. In real-world clinical settings, the accurate detection of true electrographic seizures is more important, as these events directly inform treatment decisions and therapeutic interventions.

Our model processes the full time-frequency spectrogram of each iEEG recording and identifies abnormal/ictal activity patterns that enable classification as seizure or non-seizure. While the spectrogram encodes both temporal and spectral information, our analysis in this study did not focus on isolating specific time points (e.g., immediately preceding seizure onset) that drive the classification. Instead, the model integrates abnormal activity patterns across the entire recording to reach its decision. The broader question of whether there are consistent signal patterns that are predictive of upcoming seizures but not necessarily ictal lies in the realm of seizure forecasting and is an important area for future work. Moreover, once we have supervised labeled data specifying which recordings correspond to distinct seizure onset types, it would be valuable to investigate whether different categories of non-ictal patterns preferentially precede different seizure onset types. We plan to explore this in future studies.

Hyperparameters such as learning rate, optimizer, and dropout rate were selected through manual tuning informed by initial experiments. Given the large size of the training dataset and the computational demands of training deep neural networks, more comprehensive hyperparameter optimization techniques such as grid search or Bayesian optimization were not feasible in the current study. However, future work could explore these methods to potentially further enhance model performance.

The ViT model was designed to process one iEEG channel at a time, making an independent determination about whether the input channel contained seizure activity. However, in clinical practice, epileptologists often consider information from multiple channels simultaneously when labeling a channel or determining whether an entire recording reflects seizure activity. In future work, a promising direction would be to extend the current ViT framework to incorporate multi-channel context, allowing the model to leverage inter-channel relationships and better mimic expert behavior.

In future work, it would be valuable to expand the dataset with more labeled recordings from the focal epilepsy population and improve generalizability to other epilepsy populations such as IGE and Lennox-Gastaut syndrome (LGS). These conditions exhibit distinct electrographic characteristics: IGE is typically associated with rhythmic activity < 13 Hz and generalized spike-and-wave discharges, while LGS is often characterized by low-voltage fast activity (Bullinger et al., 2025). Obtaining expert-labeled data from these populations and fine-tuning the model accordingly may enhance its ability to accurately classify electrographic seizures across diverse epilepsy types.

Currently, the ViT model is designed for binary classification, distinguishing between seizure and non-seizure activity. However, expanding its capabilities to support multiclass classification of different seizure onset patterns such as low-voltage fast activity, spike-and-wave discharges, and rhythmic delta activity, would be a valuable advancement. This could provide clinicians with more granular insights into the types of seizure onsets occurring in patients over time, aiding in both clinical decision-making and the generation of new scientific questions regarding seizure evolution and treatment response.

## Data Availability

The original contributions presented in the study are included in the article/supplementary material, further inquiries can be directed to the corresponding author.
